# Cribriform morular thyroid carcinoma: a case report with pathological, immunohistochemical, and molecular findings suggesting an origin from follicular cells (or their endodermal precursors)

**DOI:** 10.1007/s00428-023-03495-9

**Published:** 2023-01-23

**Authors:** Ana Echegoyen-Silanes, José Javier Pineda-Arribas, María Sánchez-Ares, Soledad Cameselle-García, Beatriz Sobrino, Clara Ruíz-Ponte, Magalí Piso-Neira, Emma Anda, José Manuel Cameselle-Teijeiro

**Affiliations:** 1 Pathology Department, Hospital Universitario de Navarra, Pamplona, Navarra Spain; 2 Endocrinology Department, Hospital Universitario de Navarra, Pamplona, Navarra Spain; 3 Department of Pathology, Clinical University Hospital of Santiago de Compostela, Health Research Institute of Santiago de Compostela (IDIS), Galician Healthcare Service (SERGAS), Santiago de Compostela, Spain; 4 Department of Medical Oncology, University Hospital Complex of Ourense, Galician Healthcare Service (SERGAS), Ourense, Spain; 5 Health Research Institute of Santiago de Compostela (IDIS), Santiago de Compostela, Galicia Spain; 6 Fundación Pública Galega de Medicina Xenómica, Servicio Galego de Saúde (SERGAS), Santiago de Compostela, Spain; 7 Fundación Pública Galega de Medicina Xenómica, Servicio Galego de Saúde (SERGAS), Grupo de Medicina Xenómica-Universidad de Santiago de Compostela, Centro de Investigación Biomédica en Red de Enfermedades Raras (CIBERer), Santiago de Compostela, Spain; 8 School of Medicine, University of Santiago de Compostela, Santiago de Compostela, Spain

**Keywords:** Cribriform morular thyroid carcinoma, WNT/β-catenin pathway, *APC*, CDX2, Histogenesis

## Abstract

Cribriform morular thyroid carcinoma (CMTC) is a rare malignant thyroid tumor with a peculiar growth pattern secondary to permanent activation of the WNT/β-catenin pathway. CMTC may be associated with familial adenomatous polyposis or sporadic; it shares morphological features with papillary thyroid carcinoma (PTC) and was considered a variant of PTC in the 2017 *WHO classification of tumors of endocrine organs*. The new 5th edition of the *WHO classification of endocrine and neuroendocrine tumors* considered CMTC an independent thyroid neoplasm of uncertain histogenesis. A thymic/ultimobranchial pouch-related differentiation in CMTC has been recently postulated. We, however, have used the pathological and immunohistochemical features of this case of CMTC with 2 novel oncogenic somatic variants (c.3428_3429insA, p.(Tyr1143Ter) and c.3565del, p. (Ser1189Hisfs*76) of the *APC* gene to propose an origin from follicular cells (or their endodermal precursors). As usual in CMTC, the morular component of this tumor was positive for CDX2. Given the fact that WNT/β-catenin signaling, through CDX2, activates large intestine and small intestine gene expression, we postulate that in CMTC, the tumor cells have their terminal differentiation blocked, thus showing a peculiar primitive endodermal (intestinal-like) phenotype negative for sodium-iodide symporter, thyroperoxidase, and thyroglobulin. Establishing the histogenesis of CMTC is very relevant for the development of appropriate therapies of redifferentiation, particularly in patients where the tumor cannot be controlled by surgery.

## Introduction

Cribriform morularthyroid carcinoma (CMTC) is a rare malignant thyroid tumor with a peculiar growth pattern secondary to permanent activation of the WNT/β-catenin pathway [1, 2]. In its initial description as a thyroid carcinoma associated with familial adenomatous polyposis (FAP), it was considered a distinctive follicular cell neoplasm sharing some features of papillary thyroid carcinoma (PTC) and follicular thyroid carcinoma (FTC), as well as having a tendency to multicentricity [3]. Additional pathological and immunohistochemical study of morphologically similar sporadic cases led to its consideration as the cribriform-morular variant of PTC [4]. In fact, because CMTC shares some features of PTC, this neoplasm was included as a cribriform-morular variant of PTC in the 2017 *WHO classification of thyroid tumor*s [5]. Subsequently, our group proposed to consider this tumor a distinctive thyroid carcinoma with a peculiar endodermal (intestinal-like) phenotype associated with the activation of the WNT/β-catenin signaling pathway, naming it cribriform morular thyroid carcinoma [1]. Another recent study has suggested that CMTC is not related to follicular cells, but still supports its consideration as a distinctive tumor entity [6]. According to the same authors, CMTCs lack the definitive biomarkers of thyroid follicular cell differentiation; they have little overlap with most thyroid carcinomas, and the presence of morulae could represent thymic/ultimobranchial pouch-related differentiation [6]. For these authors, the co-expression of CK5 and CD5 in the morular component along with the positivity for CDX2 (recently detected in a subset of thymic carcinomas) could indicate divergent thymic/ultimobranchial pouch-related differentiation [6]. Unfortunately, the authors do not comment on their own negative results for p63 and p40 in morular structures, which would go against their proposal given the consistent positivity for p63 and p40 described both in thyroid remnants of the ultimobranchial body (solid cell nests) and in thymic carcinomas [7–10].Finally, in the new 5th edition of the *WHO classification of endocrine and neuroendocrine tumors*, the CMTC is considered an independent thyroid neoplasm of uncertain histogenesis [2]. Understanding tumor histogenesis is essential not only to generate theoretical knowledge but also for the development of targeted treatments, for example, re-differentiation therapies in the case of some follicular lineage tumors [11].

Here we report and discuss a case of CMTC with some pathological, immunohistochemical, and molecular findings supporting a lineage from endodermal precursors of follicular cells rather than a thymic/ultimobranchial lineage.

## Materials and methods

### Immunohistochemistry and in situ hybridization

Immunohistochemistry was performed on 4-μm sections cut from both the formalin-fixed paraffin-embedded (FFPE) cell-block and the FFPE tissue block using a peroxidase-conjugated labeled dextran polymer (EnVision FLEX/HRP; Dako, Glostrup, Denmark), with 3,3′-diaminobenzidine as the chromogen (GC80611-2; Dako) in an automatic immunostainer (Autostainer Link 48; Agilent, Santa Clara, CA). The primary antibodies (clone, concentration, antigenic recovery treatment, and manufacturer) in the cell block sections were as follows: thyroglobulin (2H11 + 6E1, ready to use; Roche Diagnostics, San Cugat del Vallés, Spain), TTF1 (SPT24, pH 6; Leica Biosystems, Newcastle Upon Tyne, UK), PAX8 (MRQ-50, ready to use; Roche Diagnostics), and calcitonin (polyclonal, A0576, 1:400; Dako). For the tissue sections, the antibodies used were the following: thyroglobulin (polyclonal, ready to use, pH 6; Dako), thyroperoxidase (MoAb47, 1/50, pH 9; Dako), TTF1 (SPT24, 1/100, pH 9; Gennova, Sevilla, Spain), PAX8 (SP348, 1/100, pH 9; Gennova), cytokerantin 7 (CK7) (OV-TL 12/30, ready to use, pH 9; Dako), CK20 (Ks 20.8, ready to use, pH 9; Dako), CK5/6 (D5/16B4, ready to use, pH 9; Dako), sodium-iodide symporter (NIS) (FP5A, prediluted, pH 9; NeoMarkers, Fremont, CA, USA), galectin-3 (GAL3-3B8, 1:100, pH 9; Biocare Medical, Madrid, Spain),estrogen receptor-α (EP1/IR044IVD, ready to use, pH 9; Dako), progesterone receptor (PgR 636/IR068, ready to use, pH 9; Dako), β-catenin (β-catenin-1, ready to use, pH 9; Dako), p53 (clone DO‑7, ready to use, pH 9; Dako), CDX2 (DAK-CDX2, ready to use, pH 9; Dako), CD10 (DAK-CD10, ready to use, pH 9; Dako), CD5 (4C7, ready to use, pH 9; Dako), CD117 (CD117, 1:200, pH 9; Dako), SATB2 (EP281, 1:50, pH 9; Gennova, Sevilla, Spain), carcinoembryonic antigen (CEA) (II-7, ready to use, pH 9; Dako), and Ki-67 (MIB1, ready to use, pH 6; Dako).mRNA in situ hybridization (ISH) for thyroglobulin was performed on paraffin sections using a single-strand DNA commercial probe (CAM0016, Histosonda Thyroglobulin (Cenbimo, Lugo, Spain)), following the manufacturer’s protocol.

### Mutational analysis

#### RET/PTC rearrangement analysis

Detection of *RET/PTC* rearrangements by fluorescence in situ hybridization (FISH) was performed using the 10q11RET Break-Apart FISH Probe RUO Kit (Abbott Molecular Inc, IL, USA) following the manufacturer’s instructions. For analysis of *RET/PTC* rearrangements, at least 200 cell nuclei were scored for a split FISH signal (rearranged) in addition to an overlapping signal.

#### Real-time polymerase chain reaction

Samples containing only tumor tissue and only normal thyroid tissue were selected by macro-microdissection from 10-μm-thick sections of FFPE tissue blocks for subsequent DNA extraction as previously reported [12]. DNA was obtained using the DNA Sample Preparation Kit (Roche Diagnostics, Basel, Switzerland) and amplified by polymerase chain reaction (PCR). Normal and tumor samples were screened for *BRAF*, *NRAS*, and *KRAS* mutations using real-time PCR (Cobas *BRAF*/*NRAS* Mutation Test and Cobas *KRAS* Mutation Test; Roche), with appropriate negative and positive controls.

#### Whole-exome DNA sequencing

Whole-exome sequencing (WES) was carried out on DNA extracted from FFPE tissues of matched thyroid carcinoma and healthy tissue from the patient. DNA extraction was undertaken using the GeneRead DNA FFPE Kit (Qiagen, Germany). WES was performed using the KAPA HyperPrep Kit (Roche Sequencing Solutions, Inc.) for library preparation and KAPA HyperExome (Roche) for capturing the region of interest following KAPA HyperCap Workflow v3.2 manufacturer’s protocol. Exome libraries were sequenced on the NovaSeq 6000 (Illumina, Inc.) with 2 × 100 bp pair-end reads. The sequence reads were aligned to the human_g1k_v37 reference genome. Variant calling was performed with MUTECT2 using the tumor with matched normal mode (GATK v4.2.6.1) to obtain the tumor-exclusive variants. The analysis was restricted to the *APC*, *AXIN1*, *AXIN2*, and *CTNNB1* genes involved in the canonical WNT/β-catenin pathway. Median coverage was 454 × for the tumor and 374 × for the normal tissue. Genetic variants were described according to the Human Genome Variation Society (HGVS) (https://varnomen.hgvs.org/), and the classification of pathogenicity was performed according to the standards of pathogenicity of somatic variants in cancer [13].

### Case report

**Fig. 1:**
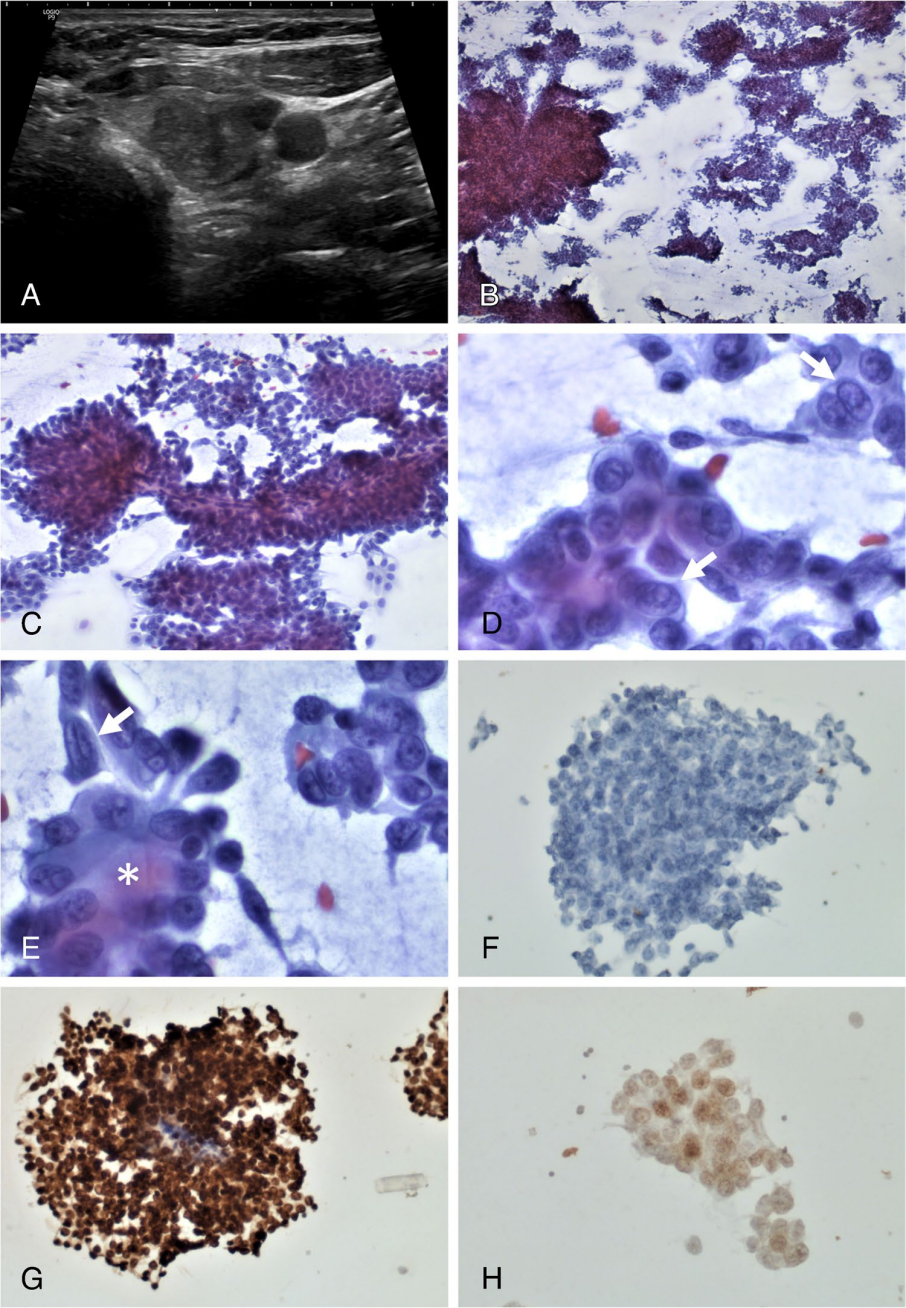
Ultrasound image showing a predominantly solid, hypoechoic thyroid nodule (**A**). The cytological smear is highly cellular and composed of monolayer sheets and papillary-like fragments (**B** and **C**). At a higher magnification, intranuclear cytoplasmic pseudoinclusions (**D**, arrows) and nuclear grooves can be seen (**E**, arrow). Follicular structures can also be detected (**E**, asterisk). Tumor cells (on cell block preparations) are negative for thyroglobulin (**F**) but strongly positive for TTF1 (**G**) and weakly positive for PAX8 (**H**)

A 29-year-old woman with normal thyroid function presented with a mass in the left thyroid lobe. She was obese (body mass index: 41) and had no personal or family data of thyroid disease or cancer. Ultrasonography showed a lobulated, predominantly solid, hypoechoic thyroid nodule (Fig. 1A), and an ultrasound-guided fine needle aspiration biopsy was performed. Based on the characteristics of the cytological smear (which included monolayer sheets, occasional papillary-like fragments, and rare tumor cells with intranuclear cytoplasmic pseudoinclusions) (Fig. 1B–E) and the immunohistochemical data of the cell-block, which was positive for TTF1 and PAX8 and negative for both thyroglobulin and calcitonin (Fig. 1F–H), a diagnosis of PTC was made (Bethesda VI).

**Fig. 2:**
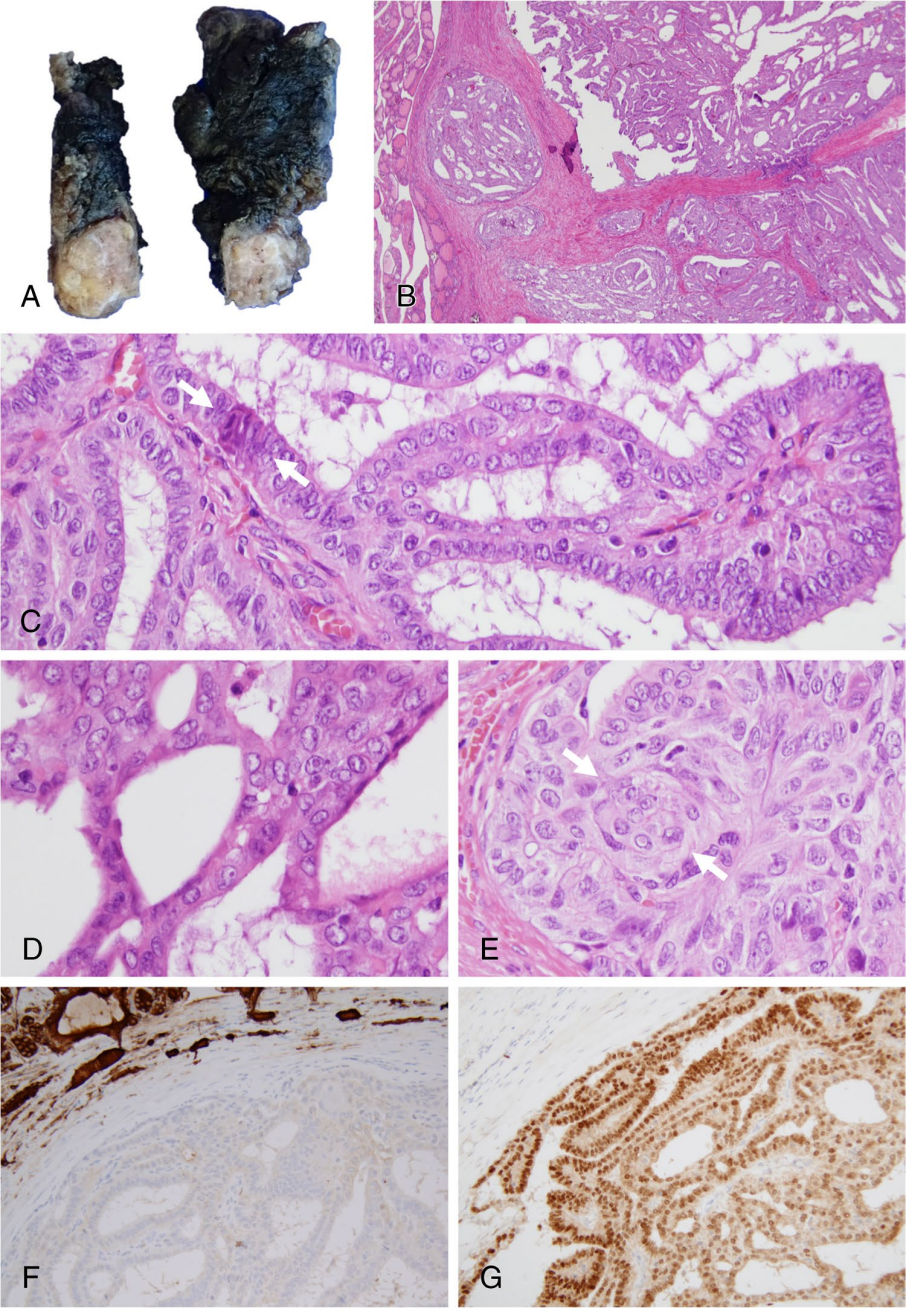
Right thyroid lobe showing the well-defined tumor with some whitish fibrous tracts inside (**A**). The tumor is well delimited by a fibrous capsule, infiltrated by small nodules (**B**). There are variable patterns of growth (**B**), including papillary areas (**C**) merging with cribriform areas (**D**) as well as a follicular pattern in other areas (**B**). Only rare, poorly developed morulae are identified with hematoxylin–eosin (**C** and **E**, arrows). Tumor cells are negative for thyroglobulin (**F**) and calcitonin but positive for TTF1 (**G**)

**Fig. 3:**
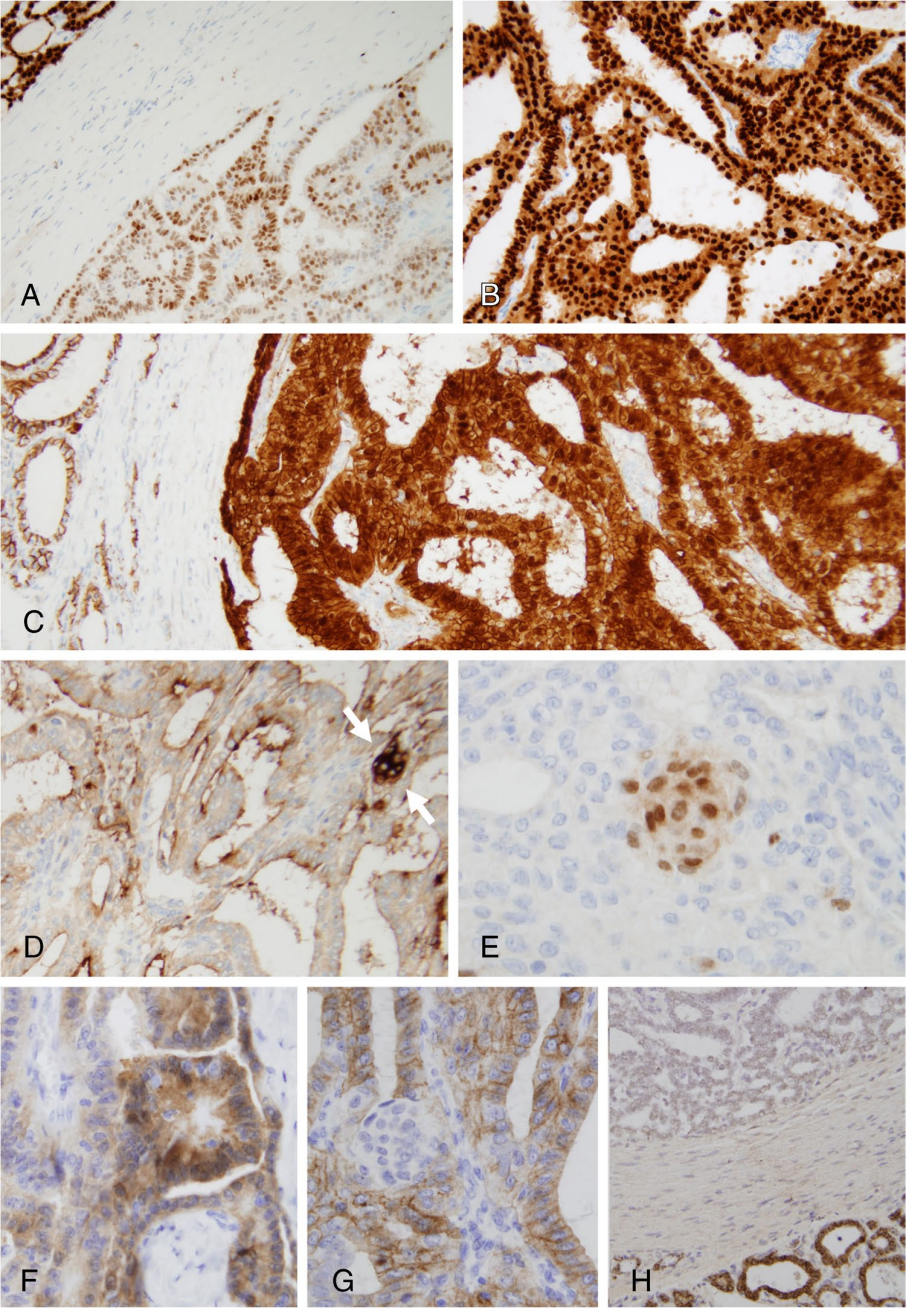
Tumor cells displayed positivity for PAX8 (**A**), cytokeratin 7, and for estrogen and progesterone (**B**) receptors, with strong nuclear and cytoplasmic positivity for beta-catenin (**C**). Morulae were easily identified by their positivity for CD10 (**D**, arrows) and CDX2 (**E**). Galectin-3 was positive in the tumor (**F**). CD117 immunostain showing positivity in the cribriform areas while the morulae are negative (**G**). mRNA in situ hybridization for thyroglobulin was negative in all tumor cells (**H**)

Total thyroidectomy was performed, and the surgical specimen showed a well-defined greyish-yellow tumor, which included some whitish fibrous tracts and measured 14 mm (Fig. 2A). Microscopically, the tumor was lobulated and well delimited by a fibrous capsule infiltrated by small nodules (Fig. 2B). The tumor had mainly a papillary growth pattern that fused with cribriform areas along with a follicular pattern (lacking colloid) in other areas. Frequently, the papillary structures were lined by tall cells with nuclear pseudostratification and nuclear features of classic PTC even in cribriform areas (Fig. 2C–E). Few and/or poorly developed morular structures were also identified (Fig. 2C and E, arrows). In the immunohistochemical study, the tumor cells were negative for thyroglobulin (Fig. 2F), thyroperoxidase, calcitonin, NIS, CK20, p63, p40, CEA, and SATB2. They were positive for CK7, TTF1 (Fig. 2G), PAX8, CD117, galectin-3, estrogen receptors, and progesterone receptors, with strong nuclear and cytoplasmic positivity for beta-catenin (Fig. 3). The Ki-67 index was 5%. The p53 protein expression pattern was wild type. ISH for mRNA thyroglobulin was negative. The presence of morulae was highlighted with immunostaining for CD10, CD5, CK5/6, and CDX2 (Fig. 3D and E). The diagnosis was cribriform morular thyroid carcinoma.

The mutational study of the tumor was negative for *RET/PTC* rearrangements and mutations of *BRAF*^V600E^, *NRAS*, and *KRAS*. Mutations of *CTNNB1*, *AXIN1*, and *AXIN2* were not found either in tumor or in normal tissue (germline). In the DNA from the CMTC, two loss-of-function variants were identified in the *APC* gene (Table 1). Neither of them was found in the DNA from the normal tissue, confirming that they were exclusive to the tumor. These two oncogenic somatic variants were not reported either in the COSMIC database or in the gnomAD v2.1.1 population database.

**Table 1:** Results of the mutational study in this case of CMTC

Gene (ID-NM)	Tumor tissue	Normal tissue
*APC* (NM_000038.6)	c.3428_3429insA, p.(Tyr1143Ter)† c.3565del, p.(Ser1189Hisfs*76)‡	WT
*CTNNB1* (NM_001904.4)	WT	WT
*AXIN1* (NM_003502.4)	WT	WT
*AXIN2* (NM_004655.4)	WT	WT
*BRAF* (NM_001374258.1)	WT	ND
*NRAS* (NM_002524.5)	WT	ND
*KRAS* (NM_004985.5)	WT	ND
p53§	WT	WT

^†^Variant allele frequency (VAF): 30%, coverage: 460 × ; ‡VAF: 60%, coverage: 225 × ; §immunohistochemical analysis of p53 protein

*WT*, wild-type; *ND*, not done

## Discussion

Here we report a sporadic case of cribriform morular thyroid carcinoma with a poorly developed morular pattern and two novel oncogenic somatic variants of the *APC* gene. This rare thyroid tumor occurs in about 16% of patients with FAP [1]. The majority of patients with thyroid cancer and FAP are women, young women with a mean age of 26 years. Curiously, the diagnosis of CMTC can precede that of FAP in up to 40% of cases [1]. In cases of FAP with CMTC, there are germline mutations in the *APC* gene that result in absence or loss of function of the APC protein [14]. Sporadic cases of CMTC result from somatic mutations in *APC* [15], *CTNNB1* [6, 16], *AXIN1* [1], and/or *KMT2D* [17], with permanent activation of the WNT/β-catenin signaling pathway. In fact, the strong cytoplasmic and nuclear staining for β-catenin reflects the activation of this signaling pathway and is the immunohistochemical hallmark of familial and sporadic forms of CMTC [1, 2, 18]. The marked predominance of CMTC in women is consistent with the strong tumor expression of estrogen and progesterone receptors and a role of sex hormones as a tumor growth promoter [1].

In the present case, despite the scarcity of morulae, the set of morphological and immunohistochemical data fits very well with the diagnosis of CMTC. Specifically, the clinical, pathological, and molecular data (two oncogenic somatic *APC* variants) are consistent with a sporadic case of CMTC. The cytological and histopathological similarities of this tumor with classic PTC are striking. These morphological similarities between PTC and CMTC, including the formation of follicular structures, suggest the possibility of a common histogenesis from follicular cells or precursors of follicular cells. The positivity for TTF1/NKX2 and PAX8 shown in the present case supports this idea [19]. The permanent activation of the WNT/β-catenin pathway induces cell proliferation and loss of differentiation through its target genes and could explain the negativity for thyroglobulin and the lack of colloid in CMTC. It is known that WNT/β-catenin signaling, via CDX2, activates large intestine gene expression at high doses and small intestine gene expression at lower doses [20]. Consequently, the follicular cells (or their precursors) in the thyroid gland of these patients, in addition to increasing their proliferation, would likely have their terminal differentiation blocked, thus showing a peculiar primitive endodermal (intestinal-like) phenotype, as has been proposed by our group [1]. Interestingly, the morular structures of CMTC are strongly positive for CDX2 [1, 2].

Galectin-3 positivity has been described in differentiated follicular cell thyroid carcinomas, especially in PTCs and in thyroid remains of the ultimobranchial body (solid cell nests) but not in non-neoplastic thyroid cells [9]. CD117 is positive in benign follicular epithelial cells and negative in malignant thyroid lesions of follicular lineage [21, 22]. CD117 along with CK5/6, p63, and p40 are usually positive markers in intrathyroid thymic carcinomas (previously known as intrathyroidal carcinoma showing thymus-like elements) [2, 10]. The CD117 protein (c-Kit) is encoded by the *KIT* gene [22]. Upon activation of CD117 by its cytokine ligand, stem cell factor, this protein phosphorylates multiple intracellular proteins that play a role in the proliferation, differentiation, migration, and apoptosis of many cell types and therefore plays an important role in hematopoiesis, stem cell maintenance, gametogenesis, melanogenesis, and in mast cell development, migration, and function [23, 24]. In our case of CMTC, positivity for CD117 (with negativity for CK5/6, p63, and p40) would identify a tumor population devoid of terminal differentiation (stem cell type); this fact also agrees with the negativity for markers of complete follicular differentiation (negativity for NIS, thyroglobulin, and thyroglobulin mRNA), as well as complete intestinal differentiation (negativity for CK20, CEA and SATB2). It is well known that WNT/β-catenin signaling is a critical component of the intestinal stem cells (ISC niche) [25]. WNT pathway activation by *APC* gene mutation and constitutive activation of β-catenin, specifically in ISCs, are sufficient to induce intestinal epithelial hyperproliferation and polyposis [25]. Therefore, in our case, it seems reasonable to propose that *APC* somatic mutations through activation of the WNT/β-catenin pathway and consequent transcription of their target genes (*MYC*, *AXIN2*, *CCND1*, and others) would block differentiation cells of follicular cells (or their neoplastic precursors), giving rise to the characteristic phenotype of CMTC.

Although some authors [6] propose that the immunohistochemical reactivity for PAX8 is weak or negative, we found positivity for PAX8 in this case using two different antibodies. The same researchers [6] postulate that morulae may represent divergent thymic/ultimobranchial pouch-related differentiation. We disagree with this idea, since morulae, in addition to showing positivity for CDX2 (an intestine-specific gene transcription factor), are also positive for CA19.9 and CD10 [1, 2]. Morular structures with the same immunohistochemical profile have been reported in pulmonary blastomas, low-grade adenocarcinomas of fetal lung type, pancreatoblastomas, and other tumors from other locations [26, 27]. In our opinion, since the CMTC lacks markers of terminal differentiation (follicular differentiation), the definitive confirmation of its cell lineage only seems possible through functional studies, for example, by blocking WNT/β-catenin signaling in primary cell cultures from samples of these tumors. It seems reasonable to suggest, however, a histogenesis from follicular cells (or their endodermal precursors) based on the following data:

- Overlapping of numerous architectural, cytological, and immunohistochemical features of CMTC with other follicular-derived thyroid tumors, especially with papillary thyroid carcinoma.
- The multifocality of CMTCs associated with germline APC gene mutation (familial adenomatous polyposis) is more consistent with an origin from thyroid follicular cells or their precursors than from some isolated thymic or branchial remnant.
- The possibility of a follicular lineage tumor with the morphological and immunohistochemical phenotype of CMTC could be explained by its molecular alterations based on the transcription of genes associated with the permanent WNT/β-catenin pathway.

Although CMTC usually has a good prognosis, some high-grade CMTCs, usually with *TERT* promoter mutations [28], have been known to behave aggressively [29–31]. This is why it is important to ascertain the exact histogenesis of CMTC. Only then can we determine the appropriate therapies of redifferentiation, especially in patients where the tumor cannot be controlled by surgery, radioactive iodine, or other treatments [32].

## Data Availability

Data are available from the corresponding author on reasonable request.
